# Analyse des Weaningprozesses bei Intensivpatienten im Hinblick auf Dokumentation und Verlegung in weiterbehandelnde Einheiten

**DOI:** 10.1007/s00063-022-00941-5

**Published:** 2022-07-11

**Authors:** Maximilian Kippnich, Tobias Skazel, Hanna Klingshirn, Laura Gerken, Peter Heuschmann, Kirsten Haas, Martha Schutzmeier, Lilly Brandstetter, Dirk Weismann, Bernd Reuschenbach, Patrick Meybohm, Thomas Wurmb

**Affiliations:** 1 Klinik und Poliklinik für Anästhesiologie, Intensivmedizin, Notfallmedizin und Schmerztherapie, Sektion Notfall- und Katastrophenmedizin, Universitätsklinikum Würzburg, Oberdürrbacher Straße 6, 97080 Würzburg, Deutschland; 2 Katholische Stiftungshochschule München, München, Deutschland; 3 Institut für Klinische Epidemiologie und Biometrie, Julius-Maximilians-Universität Würzburg, Würzburg, Deutschland; 4 Zentrale für Klinische Studien Würzburg, Universitätsklinikum Würzburg, Würzburg, Deutschland; 5 Deutsches Zentrum für Herzinsuffizienz (DZHI), Universität Würzburg, Würzburg, Deutschland; 6 Medizinische Klinik und Poliklinik I, Universitätsklinikum Würzburg, Würzburg, Deutschland; 7 Klinik und Poliklinik für Anästhesiologie, Intensivmedizin, Notfallmedizin und Schmerztherapie, Universitätsklinikum Würzburg, Würzburg, Deutschland

**Keywords:** Weaning, Langzeitbeatmung, Interhospitaltransfer, Intensivtransport, Dokumentationsqualität, Weaning, Long-term ventilation, Interhospital transfer, Intensive care transport, Documentation quality

## Abstract

**Hintergrund und Fragestellung:**

Die Entwöhnung von Beatmungsgeräten wird nicht immer auf der primär behandelnden Intensivstation abgeschlossen. Die Weiterverlegung in andere Behandlungseinrichtungen stellt einen sensiblen Abschnitt in der Behandlung und Rehabilitation des Weaningpatienten dar. Ziel der vorliegenden Studie war die Untersuchung des Überleitungsmanagements und des Interhospitaltransfers von Weaningpatienten unter besonderer Berücksichtigung der Dokumentationsqualität.

**Methodik:**

Es erfolge eine retrospektive Datenanalyse eines Jahrs (2018) auf 2 Intensivstationen eines Universitätsklinikums. Eingeschlossen wurden alle beatmeten Patienten mit folgenden Tracerdiagnosen: COPD, Asthma, Polytrauma, Pneumonie, Sepsis, ARDS und Reanimation (Beatmung > 24 h).

**Ergebnisse:**

Insgesamt konnten 750 Patienten in die Untersuchung eingeschlossen werden (Alter 64 [52, 8–76; Median, IQR]; 32 % weiblich). Davon waren 48 (6,4 %) Patienten zum Zeitpunkt der Verlegung nicht entwöhnt (v. a. Sepsis und ARDS). Die Routinedokumentation war bei den Abschnitten „Spontaneous Breathing Trial“, „Bewertung der Entwöhungsbereitschaft“ und „vermutete Entwöhnbarkeit“ ausreichend, um die Erfüllung der Parameter der S2k-Leitlinie „Prolongiertes Weaning“ adäquat zu beurteilen. Vorwiegend wurden diese Patienten mit Tracheostoma (76 %) in Rehabilitationskliniken (44 %) mittels spezialisierten Rettungsmitteln des arztbegleiteten Patiententransports verlegt (75 %).

**Diskussion:**

Die Verlegung nicht entwöhnter Patienten nach initialem Intensivaufenthalt ist ein relevantes Thema für den Interhospitaltransfer. Die Routinedokumentation eines strukturierten Weaningprozesses ist in Kernelementen ausreichend, um den Weaningprozess lückenlos zu beschreiben. Dies ist für die Kontinuität in der Weiterbehandlung dieser Patienten von großer Bedeutung.

## Abstract (en)

**Background and Objectives:**

Weaning from ventilators is not always finished in the primary intensive care unit (ICU) setting. Transfer to other treatment facilities is a sensitive stage in the treatment and rehabilitation of the weaning patient. The aim of the present study was to investigate transition management and interhospital transfer of weaning patients, with special emphasis on documentation quality.

**Methods:**

A retrospective data analysis of one year (2018) in two ICUs of a university hospital was performed. All ventilated patients with the following tracer diagnoses were included: chronic obstructive pulmonary disease (COPD), asthma, patients with multiple injuries, pneumonia, sepsis, acute respiratory distress syndrome (ARDS), and cardiac arrest (ventilation > 24 h).

**Results:**

A total of 750 patients were included in the study (median age 64 [IQR 52.8–76]; 32% female). In all, 48 (6.4%) patients were not weaned at the time of transfer (especially sepsis and ARDS). Routine documentation was sufficient for the sections “spontaneous breathing trial”, “assessment of readiness to wean” and “presumed weanability” to adequately assess the parameters of the German S2k guideline “prolonged weaning”. Predominantly, these patients were transferred with tracheostoma (76%) to rehabilitation units (44%) by specialized physician-assisted patient transport ambulances (75%).

**Discussion:**

The transfer of ventilated patients after initial ICU stay is a relevant issue for interhospital transfer. Routine documentation of a structured weaning process is sufficient in core elements to describe the weaning process. This is of great importance for continuity in the further treatment of these patients.

## Einführung

Die Entwöhnung von der Beatmung ist ein wichtiges therapeutisches Ziel im Rahmen einer Intensivtherapie. Die Entwöhnung erfolgt jedoch nicht immer auf der primär behandelnden Intensivstation. Die Gründe hierfür sind vielfältig. So kann beispielsweise die Dauer einer notwendigen Beatmung länger sein, als die erfolgreiche Therapie der Erkrankung, die eine Intensivtherapie erforderlich machte. Je nach Patientenkollektiv kann das prolongierte Weaning bis zu 30 % aller langzeitbeatmeter Intensivpatienten betreffen [2, 4, 11, 16].

In der aktuellen COVID-19-Pandemie hat die Entwöhnung vom Beatmungsgerät nochmals an Bedeutung gewonnen. Eine erschwerte Entwöhnung vom Beatmungsgerät liegt vermutlich bei über 20 % der beatmungspflichtigen COVID-19-Patienten vor [15, 19] und könnte hierdurch zur Ressourcenverknappung in der Intensivmedizin beitragen.

Von einer Langzeitbeatmung bzw. prolongiertem Weaning kann ab einer Beatmungsdauer von mindestens 2 Wochen und mindestens 2 nicht erfolgreichen Weaningversuchen gesprochen werden [13]. Der Beginn und auch der Abschluss des Weaningprozesses haben für die betroffenen Patienten eine erhebliche prognostische Bedeutung. Gelingt die Extubation während des Intensivaufenthalts nicht bzw. kommt es zu mehreren Reintubationen, verlängert sich der Intensiv- und Klinikaufenthalt deutlich, was letztendlich ein schlechteres Outcome zur Folge haben kann [5, 14].

Bei Patienten mit einer prologierten Entwöhnung vom Beatmungsgerät kann die Verlegung in eine Weaningeinheit angezeigt sein. Solche Einheiten sollten spezialisierte und zertifizierte Stationen oder Kliniken sein. Die Deutsche Gesellschaft für Anästhesiologie und Intensivmedizin (DGAI) und die Deutsche Gesellschaft für Pneumologie und Beatmungsmedizin (DGP) haben entsprechende Zertifizierungen im Angebot, sodass die Qualität der Behandlung in Weaningeinheiten nachvollziehbar dokumentiert und nachgewiesen werden kann [1, 3, 12, 20].

Werden diese Patienten von der primär behandelnden Intensiveinheit auf eine weiterbehandelnde spezialisierte Weaningeinheit verlegt, ist die Schnittstelle zwischen diesen beiden Stationen ein sensibler Abschnitt in der Behandlung und Rehabilitation des nicht entwöhnten Patienten.

Von besonderer Bedeutung hierbei sind [6, 8]:

- die strukturierte Durchführung des Weanings auf der primär behandelnden Station und dessen Dokumentation;
- die Qualität der Übergabe an die weiterbehandelnde Einrichtung;
- der Transport des nicht entwöhnten Patienten unter fortgesetzter Beatmung.

Die Datenlage zu diesen Punkten ist lückenhaft. Für eine strukturiere Übergabe ist v. a. die exakte Dokumentation der einzelnen Weaningschritte gemäß S2k-Leitlinie „Prolongiertes Weaning“ und deren Kommunikation wichtig [13]. Die Kenntnis der Patientencharakteristika ist darüber hinaus zur Planung der Vorhaltung und der Ausrüstung der bei der Verlegung eingesetzten Rettungsmittel von Bedeutung.

## Fragestellung

Ziel der vorliegenden Studie war es zu untersuchen,

1. ob die Routinedokumentation auf ausgewählten Intensivstationen eines Universitätsklinikums ausreichend ist, um alle geforderten Eckpunkte eines strukturieren Weaningprozesses zu erfassen;
2. welche Patientengruppen betroffen sind, in welche Zielkliniken sie verlegt werden und mit welchen Rettungsmitteln des arztbegleiteten Interhospitaltransfers sie transportiert werden;
3. über welchen Atemweg die Patienten zum Zeitpunkt der Verlegung beatmet werden, um die technischen Anforderungen an das Atemwegsmanagement während des Transports einzuschätzen.

Die vorliegende Studie ist Bestandteil der sog. OVER-BEAS-Studie (Optimierung der Versorgung beatmeter Patienten in der Außerstationären Intensivpflege; Förderkennzeichen 01VSF17008). In mehreren Arbeitspaketen wurden Fragestellungen zur Prozess‑, Struktur- und Ergebnisqualität bei der Versorgung heimbeatmeter Menschen bearbeitet.

## Methodik

Es erfolgte eine retrospektive Datenanalyse auf der anästhesiologischen und medizinischen Intensivstation des Universitätsklinikums Würzburg.

Die Schwerpunkte der Intensivstationen liegen in der Behandlung von Poytraumapatienten, ARDS (überregionales ECMO-Zentrum nach dem modularen Zertifikat Intensivmedizin, Deutsche Gesellschaft für Anästhesiologie und Intensivmedizin), Sepsis, kardiogenem Schock und Postreanimationssyndrom (Cardiac-Arrest-Center-Zertifikat, Deutscher Rat für Wiederbelegung).

Eingeschlossen wurden alle beatmeten Patienten mit einer invasiven oder nichtinvasiven Beatmung größer 24 h und den Tracerdiagnosen COPD, Asthma, Sepsis, Lungenversagen/ARDS, Reanimation und Polytrauma (Mehrfachnennung möglich).

Es wurde zudem eine Subgruppe gebildet, die Patienten, die bereits als außerklinisch beatmet aufgenommen wurden, umfasst.

Aus dem Patientendatenmanagementsystem (PDMS; hier COPRA, COPRA System GmbH, Berlin) der beiden Intensivstationen wurde zunächst der Beatmungsstatus aller weiterverlegten Patienten (entwöhnt, nichtentwöhnt) erfasst. Die Patienten mit dem Beatmungsstatus „nichtentwöhnt“ wurden in die weitere Analyse eingeschlossen. Warum der Entwöhnungsprozess im Einzelfall nicht erfolgreich abgeschlossen werden konnte, wurde in der vorliegenden Studie nicht explizit untersucht. Die vermutete Entwöhnbarkeit basiert auf der Einschätzung mind. eines behandelnden Arztes, ob eine Entwöhnung vom Beatmungsgerät in absehbarer Zeit als wahrscheinlich erscheint.

Zur Beurteilung der Dokumentation des strukturieren Weaningprozesses wurde die digitale Routinedokumentation der Intensivstationen betrachtet (COPRA, COPRA System GmbH, Berlin). Mittels Stichwortsuche wurden die nachfolgenden Begriffe detektiert und der Verlaufseintrag dahingehend bewertet (dokumentiert/nichtdokumentiert):

- Intubation;
- Reintubation;
- Rekanülierung;
- vermutete Entwöhnbarkeit;
- Spontaneous Breathing Trial (SBT)/Spontanatmungsversuch;
- Dekanülierung.

Die Routinedokumentation erfolgt bettseitig und in Echtzeit durch die betreuende Pflegekraft bzw. durch den behandelnden Arzt. Es ist davon auszugehen, dass die Dokumentation somit mit den unternommenen Maßnahmen übereinstimmt.

Das Entwöhnungsprotokoll (inklusive Art und Zeitpunkt der durchzuführenden obligaten und supportiven Maßnahmen) ist in einer auf die jeweilige Intensivstation abgestimmte SOP abgebildet. Diese SOP wird regelmäßig aktualisiert und den entsprechenden gültigen Leitlinien angepasst. Außerdem ist diese im PDMS hinterlegt und kann checklistenartig abgearbeitet und dokumentiert werden.

Der Interhospitaltransfer wurde hinsichtlich des Atemwegs bei Verlegung, der Art der Zielklinik bei Verlegung, des Transportmittels bei Verlegung sowie relevanter Komplikationen (letztere anhand des Protokolls für Interhospitaltransfers) untersucht. Intensivtransportwagen bzw. der Transport mit Rettungstransportwagen inklusive Verlegungseinsatzfahrzeug sind obligate arztbegleitete Patiententransporte. Die Transportärzte sind Mitarbeiter der Klinik und Poliklinik für Anästhesiologie, Intensivmedizin und Schmerztherapie und verfügen über die Zusatzbezeichnung Notfallmedizin. Im Krankentransportwagen bzw. bei Privattransporten erfolgte die Transportbegleitung durch eine Pflegekraft der aufnehmenden außerstationären Beatmungseinrichtung.

Die Daten wurden anonymisiert in ein dafür programmiertes Eingabewerkzeug eingegeben. Hierüber erfolgte die Auswertung. Das Projekt wurde der zuständigen Ethikkommission der Universität Würzburg vorgelegt und nach Beurteilung und Beratung genehmigt (Referenznummer 57/19-sc).

## Ergebnisse

Insgesamt wurden im Untersuchungszeitraum (01.01.2018 bis 31.12.2018) 750 Patienten eingeschlossen, auf die mindestens eine er definierten Tracerdiagnose zutraf. 356 Patienten (47,5 %) wurden auf der anästhesiologischen und 394 (52,5 %) auf der internistischen Intensivstation behandelt. Das Alter lag im Median bei 64 (IQR 52,8–76). 32 % der eingeschlossenen Patienten waren weiblich.

Als bereits außerklinisch beatmet wurden 12 Patienten (1,6 %) aufgenommen. 48 Patienten (6,4 %) waren zum Zeitpunkt der Verlegung nicht entwöhnt. Von der Subgruppe der als bereits außerklinisch beatmeten Patienten konnten 2 der 12 Patienten von der Beatmung entwöhnt werden und spontanatmend ohne Unterstützung weiterverlegt werden.

### Tracerdiagnosen

Bei 592 Patienten traf eine, bei 140 Patienten 2, bei 17 Patienten 3 und bei einem Patienten 4 Tracerdiagnosen bei Aufnahme zu. In Tab. 1 sind die Verteilungen der Tracerdiagnosen bei Aufnahme (aller eingeschlossenen Patienten; *n* = 750) und bei Verlegung (der nichtentwöhnten Patienten; *n* = 48) dargestellt. In der Subgruppe der als außerklinisch beatmet aufgenommenen Patienten traf bei 10 Patienten die Tracerdiagnose Sepsis zu. Zwei der 10 Patienten mit Sepsis hatten zusätzlich eine COPD. Zwei weitere Patienten der Subgruppe wurden nach Reanimation und ein Patient im Rahmen eines ARDS intensivmedizinisch behandelt.

**Table 1:** 

Tracerdiagnose	Aufnahme *n*/%	Verlegung *n*/%
COPD	93/12	6/12
Asthma	4/0,5	/
Sepsis	416/55	36/75
ARDS	58/8	16/33
Reanimation	141/19	10/21
Polytrauma	215/29	10/21

### Dokumentation des strukturierten Weaningprozesses

Die Routinedokumentation war bei den Abschnitten „Spontaneous Breathing Trial“, „Bewertung der Entwöhungsbereitschaft“ und „vermutete Entwöhnbarkeit“ ausreichend, um die Parameter der S2k-Leitlinie „Prolongiertes Weaning“ ausreichend darzustellen [13]. Die Dokumentationsqualität bei diesen Abschnitten war hoch: Spontaneous Breathing Trial 100 % (*n* = 48), Bewertung der Entwöhnungsbereitschaft 81,3 % (*n* = 39), vermutete Entwöhnbarkeit 85,4 % (*n* = 41).

Die Intubation bzw. das Vorhandensein eines Atemwegs als Startpunkt der Beatmung auf der Intensivstation war der Routinedokumentation stets zu entnehmen (100 %, *n* = 48).

Die Dokumentationsqualität „Reintubation“ (*n* = 4) und „Rekanülierung“ (*n* = 4) war in der Routinedokumentation nicht auswertbar, da die Grundgesamtheit retrospektiv nicht nachweisbar war. Eine vorübergehende Rekanülierung beispielsweise, die so nicht dokumentiert war, kann im Nachhinein auch nicht als „nichtdokumentiert“ detektiert werden.

### Atemweg bei Verlegung in ein anderes Krankenhaus (Interhospitaltransfer)

21 Patienten (43,8 %) hatten bei Verlegung ein chirurgisch angelegtes Tracheostoma, während 15 Patienten (31,3 %) ein dilatativ angelegtes Tracheostoma hatten. Jeweils 6 Patienten (12,5 %) wurden mit Endotrachealtubus bzw. NIV-Maske verlegt. Tab. 2 zeigt die Art der Zielklinik bei Verlegung.

**Table 2:** 

Art der Zielklinik bei Verlegung	*n* (%)
Grund‑/Regelversorgung	0
Schwerpunktversorgung	9 (18,8)
Maximalversorgung^a^	6 (12,5)
Rehabilitationsklinik^b^	21 (43,8)
Weaningzentrum	7 (14,6)
Außerstationäre Beatmungseinrichtung	5 (10,4)

^a^Aus Kapazitätsgründen oder zur heimatnahen Rückverlegung

^b^Fortgesetzte Akutbehandlung: Beatmungsentwöhnung (Weaningzentrum) und/oder eine neurologische Frührehabilitation (Phase B)

In Tab. 3 ist das Transportmittel bei Verlegung dargestellt.

**Table 3:** 

Transportmittel bei Verlegung	*n* (%)
Rettungstransportwagen plus Verlegungseinsatzfahrzeug	29 (60,4)
Intensivtransportwagen	14 (29,2)
Krankentransportwagen	2 (4,2)
Privattransport	3 (6,2)

Während des Interhospitaltransfers, der mit Rettungswagen plus Verlegungseinsatzfahrzeug oder Intensivtransportwagen durchgeführt wurde, traten keine relevanten transportbedingten Komplikationen (z. B. Stromausfall oder Dislokationen von Kathetern) auf.

## Diskussion

In der vorliegenden Studie wurde retrospektiv auf ausgewählten Intensivstationen eines Universitätsklinikums die Routinedokumentation im Hinblick auf die geforderten Eckpunkte des strukturierten Weaningprozesses untersucht. Der Schwerpunkt lag auf denjenigen Patienten, die während ihres Intensivaufenthalts bis zur Verlegung nicht von der Beatmung entwöhnt worden konnten. Des Weiteren wurde der Interhospitaltransfer dieser Patienten in weiterführende Behandlungseinrichtungen betrachtet.

Die Patienten, die im Untersuchungskollektiv nicht entwöhnt werden konnten, stammten überwiegend aus den Gruppen mit den Tracerdiagnosen ARDS und Sepsis. Vergleicht man die Verteilung der führenden Tracerdiagnosen des untersuchten Akutkollektivs mit denjenigen bei Aufnahme in spezialisierte Weaningzentren, fällt auf, dass sich die führenden Tracerdiagnosen unterscheiden. So gibt es Hinweise darauf, dass in Weaningzentren Patienten mit COPD, postoperativer Versorgung und Critical-Illness-Polyneuropathie (CIP) bzw. -Myopathie (CIM) den größten Anteil ausmachen [7, 13, 17]. Ursache hierfür könnte das ausgesuchte Patientengut sein, das im Studienprotokoll vordefiniert wurde. Die hier eingeschlossenen Krankheitsbilder spiegeln den Versorgungsschwerpunkt der beteiligten Intensivstationen wider.

Zur Beurteilung der wichtigen Abschnitte des strukturierten Weaningprozesses „Spontaneous Breathing Trial“, „Bewertung der Entwöhnungsbereitschaft“ und „vermutete Entwöhnbarkeit“ war die Routinedokumentation ausreichend. Es konnte außerdem gezeigt werden, dass auch die Dokumentationsqualität für diese Abschnitte sehr hoch war. Für die Parameter „Reintubation“ und „Rekanülierung“ war die Routinedokumentation nicht ausreichend. Grund hierfür ist das Fehlen der Grundgesamtheit, da das „Nichtereignis“ in der Regel nicht dokumentiert wird. Eine standardisierte Erwähnung im Entlassbrief („… eine Reintubation bzw. Rekanülierung war während des Aufenthalts nicht erforderlich …“) könnte eine mögliche Optimierung darstellen.

Fast die Hälfte der nichtentwöhnten Patienten im Studienkollektiv wurde in eine Rehabilitationsklinik verlegt, die Beatmung erfolgte größtenteils via Tracheostoma. In einer europaweiten Prävalenzstudie mit 16 beteiligten Ländern („Eurovent survey“) bezüglich Heimbeatmung war bei 17 % der außerklinisch Beatmeten der Atemweg ein Tracheostoma [9].

Der Betrachtungszeitraum der vorliegenden Studie umfasst die Akutphase der intensivmedizinischen Therapie. Es ist davon auszugehen, dass ein gewisser Anteil der identifizierten nichtentwöhnten Patienten in der weiterführenden Behandlungseinrichtung (z. B. Weaningzentrum) noch dekanüliert und vom Beatmungsgerät entwöhnt werden kann bzw. konnte [3]. Bei der Bewertung von Weaningversagen sind nicht nur medizinische, sondern auch organisatorische Aspekte zu beachten. So fand die Untersuchung auf Intensivstationen eines Akutkrankenhauses statt, die u. a. auf die Versorgung von Akutkrankheitsbildern (z. B. Polytrauma, Reanimation) spezialisiert sind und aufgrund begrenzter Bettenanzahl noch nicht vollständig entwöhnte Patienten verlegen mussten. In derartigen Fällen erfolgte nicht regelhaft eine abschließende Prüfung der Entwöhnbarkeit durch einen unabhängigen Arzt während der Initialbehandlung auf den betrachteten Intensivstationen, sondern erst im Verlauf auf der weiterbehandelnden Intensivstation. Wie den Ergebnissen zu entnehmen ist, wurde auch trotz z. T. kurzer Liegedauer regelhaft und strukturiert mit dem Weaning begonnen. Es werden weitere Studien benötigt, ob es für ausgewählte einen „idealen Verlegungszeitpunkt“ gibt und wann dieser in der intensivmedizinischen Behandlung ist.

Bemerkenswert ist in diesem Kontext die Tatsache, dass in der Subgruppe der bereits als außerklinisch beatmet aufgenommenen Patienten 2 der 12 Patienten während des akutklinischen Intensivaufenthalts erfolgreich von der Beatmung entwöhnt werden konnten. Dies könnte im Umkehrschluss bedeuten, dass auch bei chronisch beatmeten Patienten noch Weaningpotenzial vorhanden ist, nur eine regelhafte Reevaluation dieses Potenzials fehlt bzw. nur in spezialisierten Zentren ein erfolgreiches Weaning gelingen kann [3]. Diese spezialisierten Zentren sind aber nicht nur reine Weaningkliniken, sondern gerade eben auch nach DGAI bzw. DGP zertifizierte Weaningeinheiten, wie es in der vorliegenden Studie der Fall ist [1, 13].

In der Ergänzung zur S2k-Leitlinie „Prolongiertes Weaning“, die im Rahmen der aktuellen Pandemiesituation entstanden ist, wird eine neue Klassifikation von Patienten bezüglich ihres Weaningpotenzials vorgeschlagen (Kategorie A – hohes Weaningpotenzial, Kategorie B – geringes oder aktuell nicht vorhandenes Weaningpotenzial, Kategorie C – kein Weaningpotenzial) [13, 18]. Diese Kategorisierung soll Unterstützung bei der organisatorischen Planung des Weaningprozesses geben und so die intensivmedizinischen Kapazitäten und die der daran angeschlossenen Behandlungseinrichtungen optimal nutzen. Ob eine weitere Kategorisierung tatsächlich zu einer Optimierung der Kapazitätenproblematik führt, muss in weiteren Studien noch untersucht werden. Die regelmäßige Evaluation des zu entwöhnenden Patienten sowie die Routinedokumentation des strukturierten Weaningprozesses hat gerade auch in diesem Kontext einen hohen Stellenwert [10].

Der Interhospitaltransfer der nicht entwöhnten Patienten erfolgte zu ca. 90 % durch Rettungsmittel, die arztbegleitet waren und über besondere technische Ausstattung verfügen.

Der Interhospitaltransfer in Bayern erfolgt nach definierten Regeln. Das Bayerische Staatsministerium des Innern, für Sport und Integration als oberste Rettungsdienstbehörde hat hierzu ein Dispositionsschema veröffentlicht. Zunächst wird zwischen Transport mit und ohne Arztbegleitung unterschieden. Beatmete Patienten, unabhängig von Atemweg und Beatmungsmodus, werden grundsätzlich mit Arztbegleitung transportiert. Basierend auf Dringlichkeit und medizinischem Versorgungs- und Überwachungsbedarf wird durch die integrierte Leitstelle eine Vorauswahl des am ehesten geeigneten Rettungsmittels getroffen. Der begleitende Notarzt führt dann vor Transportbeginn obligat ein detailliertes und standardisiertes Arzt-Arzt-Gespräch mit Quell- und Zielklinik [21]. Dieses beschriebene Vorgehen wurde konsequent beim untersuchten Studienkollektiv angewandt.

Fünf Patienten wurden ohne Arztbegleitung verlegt. Dies waren Patienten, die von einer Intensivstation in eine außerstationäre Beatmungseinrichtung verlegt wurden. Die Transportbegleitung erfolgte hierbei durch eine Pflegekraft der aufnehmenden Einrichtung, die auch das Beatmungsgerät zum Transport zur Verfügung stellt. Diese Patienten waren alle bereits als außerklinisch beatmet aufgenommen. Das verwendete Beatmungsgerät und die Pflegekraft waren dem Patienten bekannt. In Bayern werden für Beatmungsgeräte, die nicht dem öffentlich-rechtlichen Rettungsdienst angehören, zentral Halterungen vorgehalten, die sowohl in Krankentransportwagen als auch in Rettungstransportwagen und Intensivtransportwagen angewandt werden können. Für Patienten, die in ihre außerstationäre Beatmungseinrichtung rückverlegt werden, könnte dieses Vorgehen Modellcharakter haben. Durch die Begleitung einer spezialisierten Pflegekraft der aufnehmenden Einrichtung könnten wiederkehrende Probleme im Überleitungsmanagement oder in der Verwendung unbekannter Beatmungsgeräte behoben werden.

Zusammenfassend lässt sich festhalten, dass die Routinedokumentation auf den ausgewählten Intensivstationen in den wesentlichen Punkten des strukturierten Weaningprozesses als ausreichend bewertet werden kann. Patienten, die während des Akutaufenthalts nicht entwöhnt werden konnten, stammten vorwiegend aus den Gruppen Sepsis und ARDS, was den Behandlungsschwerpunkt der Intensivstationen größtenteils widerspiegelt. Mit spezialisierten Rettungsmitteln (ITW, VEF) wurde der Großteil der nicht entwöhnten Patienten unter fortgesetzter Beatmung via Tracheostoma in Rehabilitationskliniken verlegt.

Bei der Interpretation der Ergebnisse muss bedacht werden, dass es sich bei der vorliegenden Studie um eine retrospektive Observationsstudie handelt, die in 2 Intensivstationen eines Universitätsklinikums durchgeführt wurde. Durch die Behandlungsschwerpunkte dieser Intensivstationen fand eine Vorselektion des untersuchten Patientenkollektivs statt. Patienten, die als bereits außerklinisch beatmet aufgenommen wurden, stellen im Gesamtkontext des Weanings ein besonders interessantes Kollektiv dar und sollten dementsprechend in den Fokus künftiger Untersuchungen gestellt werden. Die Qualität der Übergabe an die weiterbehandelnde Klinik ist bei der Interpretation des Gesamtprozesses ein weiterer wichtiger Aspekt, der in Folgestudien untersucht werden sollte.

## Fazit

- Die Verlegung nichtentwöhnter Patienten nach initialem Intensivaufenthalt ist ein relevantes Thema für den Interhospitaltransfer.
- Der Interhospitaltransfer dieser Patienten erfolgt vorwiegend mit spezialisierten Rettungsmitteln des arztbegleiteten Patiententransports (Intensivtransportwagen, Verlegungsarzt).
- Die Schnittstelle zwischen den verschiedenen Behandlungseinrichtungen stellt einen sensiblen Punkt in der Behandlung und Rehabilitation des beatmeten Patienten dar.
- Um das in der erstbehandelnden Intensivstation begonnene Weaning strukturiert fortführen zu können, ist die Dokumentation der verschiedenen Meilensteine im Entwöhnungsprozess essenziell.
